# Determinants of judgment and decision making quality: the interplay between information processing style and situational factors

**DOI:** 10.3389/fpsyg.2015.01088

**Published:** 2015-07-30

**Authors:** Shahar Ayal, Zohar Rusou, Dan Zakay, Guy Hochman

**Affiliations:** ^1^Baruch Ivcher School of Psychology, Interdisciplinary Center HerzliyaHerzliya, Israel; ^2^Department of Psychology, The Open University of IsraelRa’anana, Israel; ^3^Social Science Research Institute, Duke University, Durham, NCUSA

**Keywords:** biases, decision making competence, dual-process theory, individual differences, information processing style, intuitive, rational, transitivity

## Abstract

A framework is presented to better characterize the role of individual differences in information processing style and their interplay with contextual factors in determining decision making quality. In Experiment 1, we show that individual differences in information processing style are *flexible* and can be modified by situational factors. Specifically, a situational manipulation that induced an analytical mode of thought improved decision quality. In Experiment 2, we show that this improvement in decision quality is highly contingent on the *compatibility* between the dominant thinking mode and the nature of the task. That is, encouraging an intuitive mode of thought led to better performance on an intuitive task but hampered performance on an analytical task. The reverse pattern was obtained when an analytical mode of thought was encouraged. We discuss the implications of these results for the assessment of decision making competence, and suggest practical directions to help individuals better adjust their information processing style to the situation at hand and make optimal decisions.

## Introduction

Studies in the field of judgment and decision making (JDM) examine the processes underlying choice behavior, and aim to help people make decisions and adapt strategies that better fit the task at hand. Traditionally, these studies were mainly centered on aggregated behavior that reflected systematic biases and deviations from the normative benchmark (e.g., Kahneman and Tversky, 1973; Ariely, 2008). Although these studies provide valuable insights into the situational factors affecting the quality of decision making in general, it remains unclear how individual differences influence decision making quality (Franken and Muris, 2005). Ample work has shown that individual differences have pronounced effects on choice behavior (Zakay, 1990; Yechiam et al., 2005; Soane and Nicholson, 2008; Lauriola et al., 2014), risk perception (Kogan and Wallach, 1967), risk seeking and avoidance (Shaham et al., 1992) and binary guessing (Pruitt, 1961). Thus, individual differences may be crucial to a better grasp of the factors influencing performance in human decision making (Stanovich and West, 2000; Bruine de Bruin et al., 2007, 2012).

The current paper examined the effect of individual differences in information processing style and the interplay between style and the nature of the task on choice quality. Classifications of individual differences in information processing style typically categorize people as intuitive or analytical in their decision making processes (Epstein, 1994; Sloman, 1996; Evans, 2003). As recent research has shown, processing style has a marked effect on decision quality (Shiloh et al., 2002; Ayal et al., 2011, 2012; Rusou et al., 2013). For example, Stanovich and West (2000) suggested that individual differences are a key component in understanding the disparity between optimal and actual performance. Zakay and colleagues (Ayal et al., 2011, 2012; Rusou et al., 2013) found that individual differences in processing styles directly influence decision quality.

The dual-system approach and its associated models (Epstein, 1994; Sloman, 1996; Stanovich and West, 2000; Evans, 2003) posit that decision making is based on two distinct cognitive mechanisms (thinking modes)^1^. While these dual-process models may come in many flavors, they all distinguish between an intuitive mode that is assumed to be associative, quick, unconscious, effortless, and more error-prone, and an analytical mode that is assumed to be slow, conscious, effortful, and rule-based. Kahneman (2003) later suggested the terms System 1 to describe the intuitive thinking mode and System 2 to describe the analytical mode. According to the traditional dual-system approach, intuitive impressions are generated automatically, and can be overridden by conscious, effortful, deliberative reasoning. Intuitive judgments are thus considered to directly reflect impressions that are not modified by conscious deliberation (Kahneman, 2003; Hogarth, 2005; Evans, 2008). Kahneman and Frederick (2002) argued that erroneous intuitive judgments arise from biased intuitive processes, and from lax monitoring of System 2 that fails to correct these intuitive violations of normative considerations (c.f. Kahneman, 2003). It is typically assumed that people with a more analytical processing style exhibit better decision making (Kahneman and Frederick, 2002; Kahneman, 2011). In line with this claim, studies assessing performance on numerical tasks have found that analytical rule-based deliberation improves the accuracy and consistency of computations compared to reliance on intuition (MacGregor et al., 1988; MacGregor and Armstrong, 1994; McMackin and Slovic, 2000; Beilock and Decaro, 2007; Rusou et al., 2013). For instance, Mikels et al. (2013) showed that older adults experiencing a decline in analytical abilities were more prone to the ratio bias than their younger counterparts. Similarly, findings have indicated that people high in analytical processing style are less susceptible to decision biases (e.g., Banks and Oldfield, 2007; Stanovich and West, 2008; Ayal et al., 2012). The analytical thinking style was also found to be highly correlated with the Adult Decision Making Competence scale, a reliable and valid measure of decision quality (Bruine de Bruin et al., 2007, 2012; Bavol’ár and Orosová, 2015).

Despite the common belief that analytical thinking results in optimal decisions and intuitive thinking leads to biases, recent studies have found no correlation between biased decisions and intuitive thinking style (Ayal et al., 2011, 2012). In addition, recent research has also identified specific cases in which the use of analytical thinking can facilitate biased behavior (Dijksterhuis and Nordgren, 2006; Ayal and Hochman, 2009)^2^ whereas intuitive thinking can lead to more accurate and consistent decisions (Bruine de Bruin et al., 2007; Acker, 2008; Glöckner and Herbold, 2011; Usher et al., 2011).

These apparently conflicting results may stem from the dominant trend in previous research to examine information processing style (more intuitive vs. more analytical) in isolation from contextual factors such as the nature of the task. To assess decision quality, researchers frequently use decision problems that have a clear normative criterion (e.g., a criterion based on Bayes’ theorem to investigate base-rate problems; Bar-Hillel, 1980). These decision problems are rule-based in nature and hence are an advantage for analytical thinkers. However, decision problems that are more intuitive in nature might yield a different pattern of results and under certain conditions could benefit intuitive thinkers. As Sloman (2002) pointed out: “The two thinking modes are specialists at different kinds of problems. One system may be able to mimic the computation performed by the other, but only with effort and inefficiency, and even then not necessarily reliably” (p. 383).

In the current work, we suggest a more integrative approach and argue that both analytical and intuitive information processing style have the potential to lead to optimal decisions. However, the extent to which this potential can be tapped depends not only on the tendency to use a certain thinking style, but also on situational factors that prompt people to rely on a specific thinking mode (Finucane et al., 2000; Epstein, 2007). We demonstrate this flexibility in Experiment 1 by showing that decision quality can be improved by instructing participants to engage in an analytical mode of thought regardless of their information processing style. In Experiment 2, we examine whether this potential to improve decision quality depends on the compatibility between the decision maker’s dominant processing style and the nature of the task.

### The Flexibility of Information Processing Style

Individuals’ relative reliance on the analytical or intuitive thinking mode during a decision task is determined by a combination of factors, including individual differences in information processing styles, task characteristics and situational factors (Stanovich and West, 2002; Epstein, 2007). In turn, the dominant thinking mode that governs choices affects decision quality (Bruine de Bruin et al., 2007; Bavol’ár and Orosová, 2015). For example, Ayal et al. (2011, 2012) showed that people low in analytical processing style were more prone to behavioral biases. At the same time, relative reliance on processing style is also determined by situational factors. For example, time constraints (Zakay and Wooler, 1984) and cognitive load (Hoffmann et al., 2013) have been found to facilitate the use of more intuitive thinking, whereas negative affect tends to elicit the use of more analytical thinking (Schwarz and Clore, 1987). These findings suggest that over and beyond individual differences, certain situational factors can also promote the use of one thinking mode rather than another.

Experimental manipulations aimed at encouraging a more analytical or intuitive mode of thought have been found to directly influence performance on decision tasks (Dijksterhuis, 2004; Usher et al., 2011; Rusou et al., 2013). For instance, Thomas and Millar (2011) demonstrated that the framing effect was reduced when participants were encouraged to “think like a scientist.” Similarly, Hammond et al. (1987) reported that experienced highway engineers made better judgments when the properties of the task matched the mode of thinking that was used.

Based on this theoretical rationale we formulated a *flexibility hypothesis*; namely, that decision making quality is influenced by individuals’ information processing style. Specifically, we predicted that participants high in analytical information processing style should be less susceptible to cognitive biases than participants low in analytical processing. However, due to the effect of situational factors, the decision quality should be further improved even for low analytical participants by encouraging a more analytical mode of thought. We tested this hypothesis in Experiment 1.

### The Compatibility between Thinking Style and the Task at Hand

Compatibility is a crucial factor in understanding human decision making (Selart, 1997). For example, research has shown that stimulus-response compatibility plays an important role in optimizing the relationship between technology and human operators (Kantowitz et al., 1990; Kornblum et al., 1990). Reaction time and accuracy are improved when responses are compatible with the stimuli (Fitts and Seeger, 1953; Ayal and Beyth-Marom, 2014) or the information at hand (Hochman et al., 2010; Glöckner and Hochman, 2011). Similarly, compatibility between input and output is an important factor in people’s reasoning and decision making abilities (Shafir, 1995; Selart, 1996). Thus, we argue that choice quality is determined not only by the extent of reliance on each thinking mode, but also on the compatibility between the thinking style and the task at hand (Sternberg, 1999; c.f. McMackin and Slovic, 2000).

The importance of the task characteristics was first introduced by Hammond et al. (1987) who claimed that tasks are arranged on a continuum from those compatible with analytical deliberation to those compatible with intuition. According to Hammond et al. (1987), analytical tasks are characterized by a quantitative presentation, objective measures, and a readily available organizing principle. In contrast, the characteristics of intuitive tasks include high familiarity, a pictorial presentation, a subjective measure, and the unavailability of an organizing principle or algorithm to integrate cues (c.f. Epstein, 1994; Hogarth, 2005). In line with these notions, McMackin and Slovic (2000) showed that an intuitive thinking mode enhances performance on tasks that are intuitive in nature, whereas an analytical mode enhances performance on analytical tasks. However, these authors used completely different evaluation criteria for the quality of intuitive and analytical tasks which precluded testing for interactions between thinking mode and task.

This led to our *compatibility hypothesis*; namely, that decision making quality is influenced by the compatibility between the dominant thinking mode used for the decision and the nature of the task. Therefore, we predicted an interaction effect such that a manipulation that induces an analytical mode of thought should lead to higher decision quality when the nature of the task is analytical, and an intuitive mode of thought should lead to higher decision quality when the nature of the task is intuitive. We tested this hypothesis in Experiment 2.

## Experiment 1

Experiment 1 was designed to examine how individual differences in processing styles affect the quality of people’s decisions. In addition, we tested our *flexibility hypothesis* that this effect is sensitive to manipulations promoting a specific thinking mode. Based on previous findings (Ayal et al., 2011, 2012) and specifically the analytical nature of the task, we hypothesized that people high in analytical processing style would be more calibrated to normative considerations (i.e., less prone to cognitive biases in their decisions) than people low on analytical style. However, based on the same findings and the nature of task, we predicted no correlation between intuitive thinking style and calibration. Moreover, according to our flexibility hypothesis, we also expected that encouraging an analytical thinking mode (versus an intuitive mode) would further improve decision making.

### Method

#### Participants

Eighty-one undergraduate students from the Interdisciplinary Center (IDC) Herzliya (41 female, Mean age = 25.9 years, SD = 2.71) volunteered to participate in the study as part of their academic requirements in return for credit hours. All participants were native Hebrew speakers. The participants signed a consent form at the beginning of the experiment, and were debriefed at the end of the third stage.

#### Design and Procedure

We employed a 2 (processing style: analytical and intuitive) × 2 (mode of thought: analytical vs. intuitive) between-participants design. The experimental procedure was comprised of three stages. The first stage consisted of the 24-item Rational Experiential Inventory (REI) questionnaire (Pacini and Epstein, 1999) translated into Hebrew and validated in previous studies (Ayal et al., 2011, 2012). The REI is a self-report inventory that assesses individuals’ tendencies to include analytical and intuitive considerations in their decision making processes. It consists of two unipolar scales (12 items each) which rank participants on two dimensions of information processing style. The first scale measures engagement in and favorability of cognitive activities and corresponds to a rational-analytic information processing style (e.g., I have a logical mind). The second scale measures engagement in and favorability of experiential activities and corresponds to an intuitive processing style (e.g., When it comes to trusting people, I can usually rely on my gut feelings). Participants are required to state how true each statement is for them, on a scale from 1 (Definitely False) to 5 (Definitely True). Research has shown that the internal consistency reliability coefficient for each scale is high (usually above 0.85) whereas the correlation between them is small and negligible (Pacini and Epstein, 1999; Ayal et al., 2011). Thus, the REI is assumed to support Epstein’s (1994) claim of two independent information processing systems.

The second stage of the experiment consisted of the mode of thought manipulation. Participants were randomly assigned to one of two experimental conditions (analytical vs. intuitive mode of thought). Based on Usher et al.’s (2011) “declared” procedure, mode of thought was manipulated by informing participants about the “proven benefits” of decisions based on a specific thinking mode. Participants in the intuitive group were told that “Research has shown that the best decisions are the ones made using intuition” and were encouraged to base their evaluation on their “gut-feeling” and general impressions. Participants in the analytical group were told that “Research has shown that the best decisions are the ones made using logic and analytical thought” and were encouraged to think carefully and logically about their choices (Usher et al., 2011). No time limitations were imposed.

Finally, to assess decision quality, the third stage was made up of six prototypical questions used to examine adherence to biased thinking. All questions were presented in a random order. The six biases included in this stage were the ratio bias (Denes-Raj et al., 1995), proportion dominance (Fetherstonhaugh et al., 1997), irrational diversification (Ayal and Zakay, 2009), debt-account aversion (Amar et al., 2011), the gambler’s fallacy (Kahneman and Tversky, 1972; Tversky and Kahneman, 1974), and the “hot hand effect” (Gilovich et al., 1985). For instance, one question examined the ratio bias; i.e., the tendency to judge a low probability event as more likely when presented as a large numbered ratio (e.g., 9/100) than as a smaller-numbered but equal or better ratio (e.g., 1/10). This effect is attributed to a tendency to focus on the frequency of the numerator instead of the overall proportion (Miller et al., 1989; Kirkpatrick and Epstein, 1992). In this case, a choice that reflects a preference for the 9/100 ratio over the 1/10 ratio was classified as a bias. All questions were adapted from their original papers. The full questionnaire and its scoring method are presented in Appendix A. The Ethics Committee of IDC approved this study.

### Results

Before testing our hypotheses, we assessed the reliability and validity of the REI questionnaire. First, we calculated the reliability of the REI scale using Cronbach’s alpha coefficient. As predicted by the dual system approach, we found high internal consistency for the analytical (Cronbach’s α = 0.88) and the intuitive (Cronbach’s α = 0.90) scale. In addition, no correlation was found between the two scales (*r* = -0.008, *p* = 0.946).

Next, we examined whether participants exhibited judgment biases. To do so, we coded each response that was predicted by the normative solution as “1,” and each response that was predicted by the corresponding bias as “0.” Then, we calculated the average percentage of biases for each participant according to the formula (number of biased responses/6) * 100 (Cronbach’s α = 0.29). This analysis revealed that on average, participants exhibited biased thinking 52.47% of the time (SD = 21.43).

To test the relationship between the thinking mode and quality of decisions, we used a simultaneous regression analysis. Specifically, the analytical and intuitive scales (as a continuous variable) and the mode of thought (as a binary variable, with analytical mode coded as 0 and intuitive mode coded as 1) were entered into the model to assess the effect of these factors on overall bias adherence as a dependent variable. The results of this analysis are summarized in **Table 1**. As can be seen from the table, the overall model was significant [adjusted *R*^2^ = 0.10, *F*(3,77) = 4.121, *p* < 0.01]. Mode of thought was positively but marginally associated with biased results (*b* = 0.216, *p* = 0.055). Since an analytical mode of thought was coded as 0 and intuitive as 1, this result suggests that in this task, asking people to provide a more analytical decision resulted in fewer biases than asking them to provide a more intuitive response.

**Table 1:** Relationship between mode of thought and thinking styles (REI) and overall bias adherence as the dependent variable.

	Regression model		
Variables	Beta (standardized coefficients)	*t*	Significance
Mode of thought	0.216	1.949	0.055
Analytical scale	-0.227	-2.135	0.036
Intuitive scale	0.125	1.129	0.226
**Model fit**			
Adjusted *R*^2^	0.105		
*F*	4.121**		
Df	3, 77		


*N* = 81, **p < 0.01.

**FIGURE 1:**
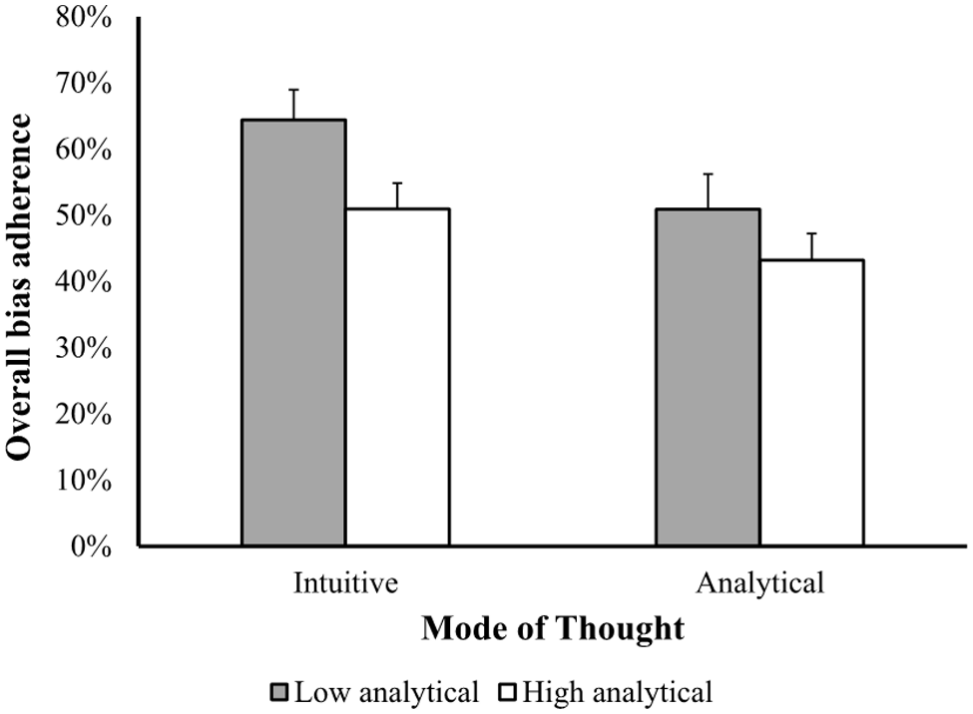
**Effects of individual differences in analytical information processing style and manipulated thinking mode on susceptibility to biases**.

In line with previous research (Ayal et al., 2011, 2012), there was a negative relationship between analytical thinking style and adherence to biases (β = -0.227, *p* < 0.05), and no association between biased behavior and intuitive thinking style (β = 0.125, *p* = 0.26, n.s.). Based on these findings, in the following analyses we focused only on the analytical scale, and examined adherence to biases separately for participants who were found to be high or low on the analytical scale (based on the median split) as a function of the mode of thought. The results of this analysis are presented in **Figure 1** and summarized in **Table 2**. As can be seen in the figure, participants high in analytical processing style exhibited fewer biases (*M* = 46.7%, SD = 18.2) than low analytical participants (*M* = 58.1%, SD = 23). Similarly, participants in the analytic mode of thought condition were less prone to biases (*M* = 46.7%, SD = 21.1) than participants in the intuitive mode of thought condition (*M* = 58.3%, SD = 20.3). A two-way ANOVA revealed a significant effect for level of analytical processing style [*F*(1,77) = 5.480, *p* < 0.03], as well as for mode of thought [*F*(1,77) = 5.53, *p* < 0.03]. No significant interaction was found between the two factors [*F*(1,77) = 0.408, n.s.].

**Table 2:** Overall mean percentage of bias adherence in the two mode of thought conditions of Experiment 1, as a function of the level of analytical processing style.

	Analytical processing style		
Mode of thought	Low	High	Overall
Analytical	50.87 (23.22)	43.18 (19.01)	46.74 (21.15)*
Intuitive	64.39 (21.39)	50.93 (16.64)	58.33 (20.32)*
Overall	58. 30 (23.01)*	46.66 (18.18)*	52.47 (21.43)

SD appears in parentheses. *Represents a significant difference (*p* < 0.05).

This pattern of results supports our hypothesis that individual differences in analytical processing style affect the quality of decisions, such that low analytical participants are more prone to cognitive biases than high analytical participants. In addition, the results also support the flexibility hypothesis by showing that inducing an analytical mode of thought can further improve decision quality.

## Experiment 2

The results of Experiment 1 supported the flexibility hypothesis and suggested that adherence to biased behavior depends on individuals’ analytical processing style, but can also be improved when an analytical mode of thought is induced. Experiment 2 was designed to test the *compatibility hypothesis* and examine whether the effect of thinking mode on decision quality is contingent on the nature of the task (Rusou et al., 2013). Specifically, we hypothesized that the advantage of analytic thinking in Experiment 1 was due to the choice of task, which was analytical in nature. In Experiment 2 we examined the effect of the interaction between thinking mode and the nature of the task on decision quality. Based on the theoretical framework presented above, we predicted that inducing an analytical thinking mode would improve performance on an analytical task, whereas encouraging an intuitive mode of thought would improve performance on a task that was more intuitive in nature.

For the analytical task, we used arithmetic multiplication. This task was chosen because it is abstract and symbolic, and can be evaluated objectively by applying well-defined mathematical rules to find or estimate the answer. Hence, it is compatible with the characteristics of analytical thinking as logical (rule-based), abstract (i.e., encoding reality in abstract symbols, words, and numbers) and emotion-free (Payne et al., 1993). For the intuitive task, we used a task that required participants to assess faces on repeated trials. Previous studies have characterized impression formation from facial appearance as a holistic process that occurs rapidly, effortlessly, and spontaneously (Todorov and Uleman, 2003). Therefore, this kind of task is considered intuitive in nature (c.f., Epstein, 1994; Lieberman et al., 2002; Hogarth, 2005; Evans, 2008; Rusou et al., 2013).

To determine decision quality, we calculated the number of transitivity violations made by the participants. The principle of transitivity implies that for any three alternatives (A, B, C), if A is judged as better than B, and B is judged as better than C, then A should also be judged as better than C (von Neumann and Morgenstern, 1944). Hence, if there is no error in decision making (or if it is very low), individuals will evaluate the different alternatives in a consistent way every time and will exhibit no (or very few) violations of transitivity in this pairwise choice paradigm (Lee et al., 2009). We chose transitivity as a dependent measure, since it is defined as one of the foundations of rationality. As stressed by Gilovich and Griffin (2010): “Transitivity is one of the axioms and principles that one’s choices must follow in order to ensure that one maximizes overall utility.” In addition, since this criterion could be used to evaluate both the analytical and intuitive task, it enabled us to test our compatibility hypothesis directly.

### Method

#### Participants

Forty undergraduate students (32 females, Mean age = 22.6 years, SD = 1.84) from the IDC Herzliya, Israel volunteered to participate in this study as part of their academic requirements in return for credit hours. All participants were native Hebrew speakers. The participants signed a consent form at the beginning of the experiment, and were debriefed at the end of the third stage.

#### Stimulus Material

**FIGURE 2:**
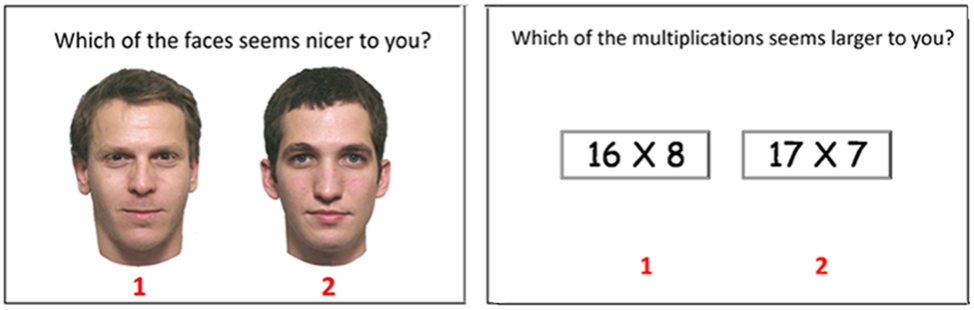
**An example of one pair of stimuli from each of the tasks used in Experiment 2**.

Evaluations of arithmetic multiplications were examined on eight multiplications in which a single-digit number was multiplied by a two-digit number (in each pair). Using all eight multiplications, 28 pairs were presented to the participants by creating all possible pairwise combinations of the multiplications (8X7/2 = 28). Within each pair, the multiplications were centered horizontally in the middle of the screen, with the digit “1” displayed under the left-hand multiplication and the digit “2” under the right-hand multiplication (see **Figure 2** for an example). The choice criterion “Which of the multiplications looks larger to you?” was presented above each pair in the upper middle part of the screen. The term “looks” was used to avoid directing participants to any specific mode of thought.

Similarly, face assessments were examined by using eight color photographs of male faces. Twenty-eight pairs were formed by creating all possible pairwise combinations of the faces (8X7/2 = 28). As with the multiplications, within each pair, the faces were centered horizontally in the middle of the screen, with the digit “1” displayed under the left-hand face and the digit “2” under the right-hand face (see **Figure 2** for an example). The choice criterion “Which of the faces looks nicer to you?” was presented above each pair in the upper middle part of the screen.

#### Design and Procedure

Participants were randomly assigned to one of four conditions, in a 2 (thinking mode: intuitive vs. analytical) × 2 (choice task: multiplications, facial impression formation) between-subjects design.

The experimental procedure was made up of two stages. The first stage consisted of the mode of thought manipulation. This stage was identical to the mode of thought manipulation in Experiment 1. The second stage consisted of the multiplication/face assessment task. In both conditions, participants were told “In a moment, you will be presented with pairs of multiplications/photographed faces. Each individual multiplication/face might appear several times; however, each combination of multiplication/faces will be presented only once. Within each pair, you will be asked to choose the multiplication that looks larger/face that looks nicer to you, by selecting the number displayed under that multiplication/face.” Following the initial instructions, the 28 pairs of multiplications/faces were presented to participants in a random order.

In the analytical condition, after the presentation of each pair, and before making their choice, participants were presented with the following two questions (one for each pair): (a) “Please specify your reasons for evaluating how large the multiplication in 1 is [nice the Face in 1 is]”; and (b) “Please specify your reasons for evaluating how large the multiplication in 2 is [nice the Face in 2 is].” Participants were required to write their responses in a text box. The questions were presented one at a time, in the same order on each trial. These questions were presented to make sure that participants in this condition would use more analytical processing (Wilson and Schooler, 1991).

Each selection of a multiplication/face was followed by a blank screen, and then the next pair of multiplications/faces appeared. Each participant was tested individually in a small room. After responding to all 28 pairs, participants were debriefed and thanked. The Ethics Committee of the IDC approved this study.

### Results and Discussion

The number of transitivity violations was calculated for each participant by counting the number of three-way cycles of transitivity violations (e.g., for each sub-group of three multiplications/faces x, y, and z, x ≥ y, y ≥ z, and z ≥ x) committed by participants (for a detailed explanation of this method, see Lee et al., 2009).

**FIGURE 3:**
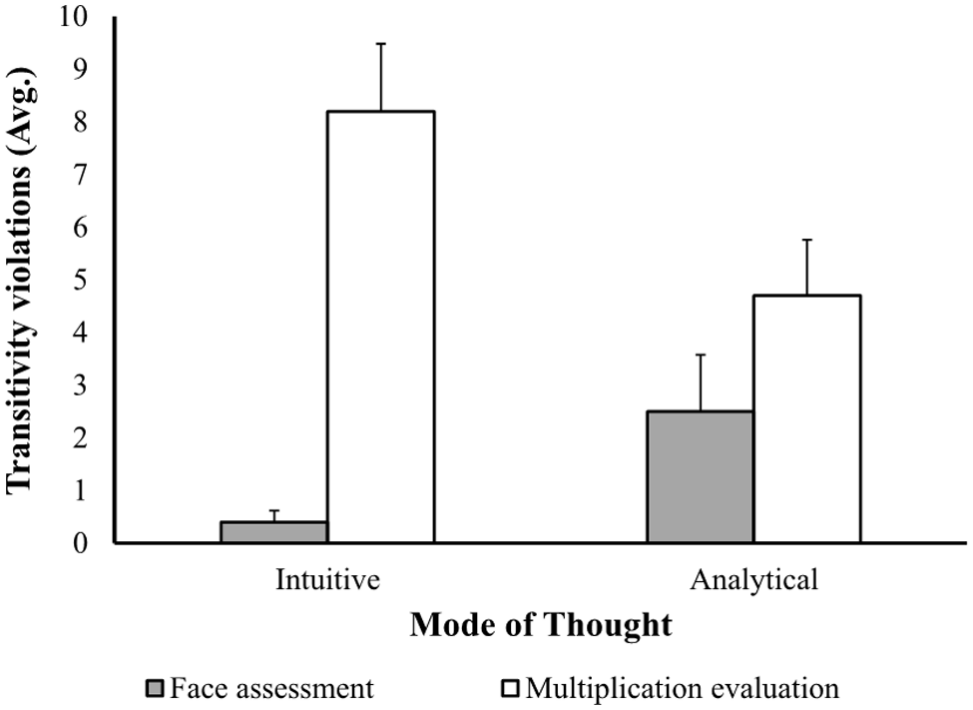
**The mean number of transitivity violations as a function of task and mode of thought**.

**Figure 3** depicts the mean number of transitivity violations in the multiplication evaluation and face assessment tasks as a function of mode of thought. In line with the compatibility hypothesis, a 2 (thinking mode: analytical vs. intuitive) × 2 (task type: multiplications vs. faces) between-subjects ANOVA on the transitivity violations showed a significant interaction effect between thinking mode and task type [*F*(1,38) = 7.88, *p* < 0.01]. Planned comparisons further revealed that in the analytic (multiplication evaluation) task, participants who were encouraged to adhere to an analytic mode of thought committed significantly fewer transitivity violations (*M* = 4.7, SD = 3.36) than those who were encouraged to think intuitively [*M* = 8.2, SD = 4.05; *t*(18) = 2.10, *p* < 0.05]. By contrast, in the intuitive (face assessment) task, participants who were encouraged to think intuitively committed significantly fewer transitivity violations (*M* = 0.4, SD = 0.7) than participants who were encouraged to use a more analytical mode of thought [*M* = 2.5, SD = 3.4; *t*(18) = 1.90, *p* < 0.05]^3^. These results are summarized in **Table 3**.

**Table 3:** Mean transitivity violations as a function of the task and mode of thought in Experiment 2.

	Task		
Mode of thought	Facial impressions	Math multiplications	Overall
Analytical	2.5 (3.40)*	4.7 (3.37)*	3.6 (3.48)
Intuitive	0.4 (0.70)*	8.2 (4.05)*	4.3 (4.9)
Overall	1.45 (2.62)	6.45 (4.05)	3.95 (4.21)

SD appears in parentheses. *Represents a significant difference (*p* < 0.05).

Importantly, since mathematical multiplications are rule-based, it was important to control whether this measure of transitivity was related to accuracy. Hence, we also calculated the percentage of correct choices on this task. This analysis showed that accuracy paralleled the pattern obtained by using transitivity violations. A *t*-test for independent samples indicated that participants who were encouraged to adhere to an analytical mode of thought were significantly more accurate in their mathematical choices (*M* = 0.68, SD = 0.18) than those who were encouraged to think intuitively [*M* = 0.56, SD = 0.13; *t*(18) = 1.7, *p* = 0.05, one-tailed].

These findings support our compatibility hypothesis that decision quality depends on the compatibility between the dominant thinking mode used for making the decision and the nature of the task at hand. Inducing a more analytical thinking mode led to more consistency (and thus to better decisions) when the task required more analytical skills, but to less consistency when the task required more intuitive skills. Inducing an intuitive mode of thought led to more consistency when the task required intuition and to less consistency when it required more analytical skills. In both cases, the improvement in choice was observed in the compatible conditions when there was a good fit between the thinking mode and the nature of the task.

## General Discussion

Proponents of dual-process decision models suggest that information processing style, an individual tendency which determines to what extent decisions rely on intuitive and analytical processes (Denes-Raj et al., 1995), is a main factor in decision quality. Traditionally, dual-process decision models have assumed that biased behavior stems from erroneous intuitive processes and a failure of analytical processes to govern behavior (e.g., Kahneman, 2003, 2011). However, recent research has found no correlation between intuitive processing style and biased behavior (Ayal et al., 2011, 2012). Moreover, it has been shown that under certain conditions, intuitive thinking can lead to decisions that are better than decisions based on analytical thinking (Wilson and Schooler, 1991; Dijksterhuis et al., 2006; Rusou et al., 2013).

To account for this apparent contradiction between empirical findings and the theoretical claims of the dual system approach, we put forward a flexible-compatible information processing style framework which posits that decision quality is not merely the result of individual differences in information processing style. Rather, the dominant style of thinking (that is, the type of processes activated during decision making) is determined by both personal tendencies and situational factors. Both intuitive and analytical processing styles can lead to optimal decisions. However, this potential is flexible. To what extent it is realized depends considerably on the compatibility between the dominant thinking style and the characteristics of the task. Analytical thinking (which is dominant among individuals high on this processing style or under situational factors that encourage this processing style) will lead to better performance on tasks that are analytical in nature. By contrast, on tasks which require more intuitive skills, an intuitive processing style will be more advantageous.

Two experiments were presented to examine this framework. In Experiment 1, we demonstrated that the effect of information processing style is flexible and can thus be modified. As in previous research, we found that individuals high in analytical processing style were less prone to biases than individuals low in analytical processing. However, as suggested by our flexibility hypothesis, encouraging an analytical mode of thought further improved decision quality in individuals both low and high in analytical information processing style. In addition, similar to previous studies (Ayal et al., 2011, 2012), in Experiment 1 there was no correlation between the intuitive scale of the REI and decision quality. This latter finding supports our compatibility assertion, as it suggests that intuitive processing style might not play a major role in analytical tasks. It also suggests that susceptibility to cognitive biases in well-known JDM tasks (e.g., the Ratio Bias, the Gambler’s Fallacy) mainly depends on the level of analytical thinking.

Further direct evidence for the compatibility hypothesis was found in Experiment 2, where participants were given either an analytical or an intuitive task. Improvement in decision quality (indexed by transitivity of choice behavior) was obtained by encouraging an analytical or intuitive mode of thought, depending on the nature of the task. That is, encouraging an intuitive mode of thought (compared to analytical) led to better performance on the intuitive-pictorial task but hampered performance on the analytical-numerical task. The opposite pattern was obtained when we encouraged an analytical thinking mode. Presumably, when the nature of the task requires intuitive skills, analytical and deliberative thinking may reduce decision quality (Wilson and Schooler, 1991; Dijksterhuis et al., 2006; Rusou et al., 2013). However, using intuition and relying more on gut feelings in such cases can lead to better choices (see also McMackin and Slovic, 2000; Rusou et al., 2013).

The current results also have important methodological implications. Decision-making research is based on comparing human choice to a normative benchmark derived from formal statistics and probability theories, logical thinking and rationality (for a review see Kahneman et al., 1982; Gilovich et al., 2002). As a result, most tools that are aimed at evaluating decision quality are based on analytical problems with an optimal solution that can be solved by implementing a normative model (e.g., A-DMC, Frederick, 2005; Peters et al., 2006; Bruine de Bruin et al., 2007). Our results suggest that measures of decision making competence should not be solely based on analytical tasks that require logical or numerical skills, but also on intuitive tasks. Different types of tasks may better represent the wide range of decision tasks faced in real life situations.

Finally, from a practical point of view, our framework suggests that individuals and organizations should pay special attention not only to how decisions are made (i.e., based on analytical or intuitive processes), but also what type of thinking is required for the specific tasks at hand. Encouraging compatibility between the dominant thinking mode and the nature of the task is a crucial aspect of decision making quality. This is true for recruiting processes, as well as for the design of a working environment that can encourage a compatible thinking mode for each task. Most importantly, people should be constantly reminded that decision making style is flexible, and that an adaptive decision maker should have more than one mode of thought in her decision making toolbox. Thus, with proper training, policy makers can help people adjust their thinking style to the situation at hand, strengthen the weaker aspects of their thinking style, and realize their full potential to make optimal decisions.

### Limitations and Future Directions

This study examined the effects of processing style on the quality of judgment and decisions, both by measuring individual differences in the tendency to think more analytically or more intuitively and by explicitly manipulating the dominant mode of thought. However, the measure we used to assess individual differences in thinking styles (REI) is only one of several (see for example, Scott and Bruce, 1995) and there are other ways of inducing a specific thinking mode (e.g., Pham, 2004; Lee et al., 2009). Similarly, our dependent measure assessing the quality of decisions was based on a set of well-known decision making tasks that cover different features of decisions. However, the internal consistency between them was low, which might limit the generalizability of our results. Thus, in future research a more standardized tool should be used to assess well-defined facets of decision making (e.g., A-DMC, Frederick, 2005; Peters et al., 2006; Bruine de Bruin et al., 2007). In addition, flexibility of processing style was only demonstrated for the analytical scale. Examining the flexibility of the intuitive style would thus be an important step for future research. For example, Experiment 2 could be replicated with the addition of measuring individual differences in processing style to examine whether encouraging an intuitive mode of thought could further improve performance on intuitive tasks. It would be useful to include control groups that are not administered mode of thought manipulations.

Second, the sample size in Experiment 2 was relatively small. Although the findings for one condition were replicated, and the overall pattern of results adhere to previous research (e.g., Rusou et al., 2013), it is important to replicate the current findings with larger sample sizes. Third, Experiment 2 implemented both intuitive and analytical tasks that were compared using the same normative criterion (i.e., transitivity). However, the higher number of transitivity violations under the math multiplication task compared to the facial impression task (across the manipulation conditions) raises a concern that these two tasks differed in difficulty level. Thus, a challenge for future research is to identify tasks which require either analytical or intuitive skills that are balanced for difficulty level.

Finally, the majority of our participants in Experiment 2 were females (80%), a fact that might limit the generalizability of our results to more heterogeneous samples. For instance, some research that has investigated the relationship between gender and information processing styles has found that females may be higher in intuitive thinking whereas males may be higher in deliberative thinking (e.g., Pacini and Epstein, 1999; Gigerenzer et al., 2014). In contrast, other studies found no gender differences in thinking styles (Delaney et al., 2015), and some have pointed out that females tend to perform as well or even better than males on mathematical tests (Hyde et al., 1990; Bridgeman and Wendler, 1991; Stout et al., 2011). Thus, the effect of gender on the relationship between thinking mode and performance on different types of tasks should be further examined. It would be useful to test whether gender groups differ not only in their dominant thinking mode, but also in the malleability of this mode, and its adaptation to different types of decisions.

## Conflict of Interest Statement

The authors declare that the research was conducted in the absence of any commercial or financial relationships that could be construed as a potential conflict of interest.

## Footnotes

1 Several scholars have questioned the theoretical coherence as well as the evidence for the existence of two systems and suggested alternatives to the two-system approach (e.g., Reyna, 2004; Keren and Schul, 2009; Strough et al., 2011). For instance, Wastell (2014) argued that human reasoning consists of a multitude of modules that interact via dynamical emergent processes based on information input and output requirements. Kruglanski and Gigerenzer (2011) proposed a unified theoretical approach to both intuitive and deliberative judgments, and claimed that both are rule-based and the issue is how the rules are selected. They suggested that rules are selected by the task itself and the individual’s memory constraints (for a recent response see Evans and Stanovich, 2013).

2 Several researchers have reported difficulties in directly replicating the effect of the superiority of unconscious intuitive thinking as reported by Dijksterhuis and his colleagues; other studies have shown that the superiority of unconscious intuitive thinking is in fact contingent on other variables (for a review see Acker, 2008; Payne et al., 2008; Nieuwenstein et al., 2015).

3 This pattern of results was replicated in another experiment that only implemented the face assessment task (*n* = 20). Face assessments were examined with nine color photographs of male faces. 36 pairs were formed by creating all possible pairwise combinations of the faces (9X87/2 = 36). The experimental procedure was identical to the procedure in Experiment 2. Participants in the intuitive mode of thought condition were more consistent between choices (*M* = 0.8, SD = 1.23) than participants in the analytical mode of thought condition (*M* = 2.2, SD = 2.15). A *t*-test for independent samples indicated that this difference was significant [*t*(18) = -1.78, *p* < 0.05, one-tailed].
