# Supplementary material for: Determinants of judgment and decision making quality: the interplay between information processing style and situational factors
Source: Front Psychol. 2015 Jul 30;6:1088. doi: 10.3389/fpsyg.2015.01088 (PMC4519675; doi:10.3389/fpsyg.2015.01088)
Supplement: Supplementary file 1 [file Data_Sheet_1.DOCX]

Appendix A

*The six questions used in Experiment 1 (translated from Hebrew) were designed to examine biased behavior. For the analyses, the normative response (presented in parentheses) was coded as 0 and biased response was coded as 1.*

**Ratio Bias (Danes-Raj et al., 1995)**

Consider 2 urns filled with marbles.

| Urn A | Urn B |
| --- | --- |
| 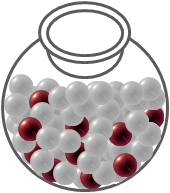 | 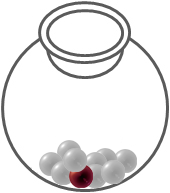 |
| contains 100 marbles, 9 of  which are red | contains 10 marbles, 1 of  which is red |

The experimenter will draw a marble from one of the urns. If a red marble is drawn, you will win a monetary prize. Which urn do you choose?

1. Urn A 2. Urn B

(Urn B)

**Proportion Dominance (Fetherstonhaugh et al., 1997)**

Imagine that you are the chairperson on the board of "Science For Life", a charitable foundation in charge of distributing large sums of money to research institutions that develop treatments for serious diseases. Medical Institutions (X) [Y] {Z} have developed a treatment for Diseases (A) [B] {C} and now request $10 million from Science For Life. Last year, people with diseases (A) [B] {C} did not have access to this treatment, and (15,000) [160,000] {290,000} died from the diseases.

Given Science For Life’s shrinking budget, what is the minimum number of lives this treatment would have to save next year in order for Medical Institutions (X) [Y] {Z} to merit funding?

Treatment A_____ Treatment B_____ Treatment C_____

(X = Y = Z)

**Irrational Diversification (Ayal & Zakay, 2009)**

Imagine five stacks of lottery tickets. Each ticket has the numbers 1 to 49. A computer will randomly select five different numbers from this range. In order to win a monetary prize, you are required to guess the numbers that will come up. To take part in the bet, please select method A or method B below:

| Method A | Method B |
| --- | --- |
| 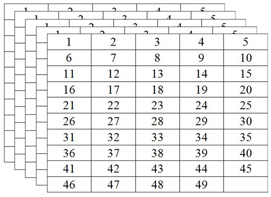 | 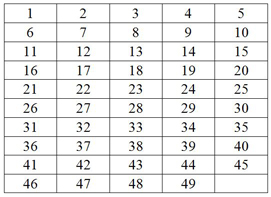 |
| Pick five tickets and mark five numbers on each of them. If you mark at least one of your tickets with the five numbers that are randomly selected in the lottery, you win | Pick one lottery ticket and mark six numbers from the entire range. If you mark the five numbers that are randomly selected in the lottery, you win |

Which of the two methods do you prefer?

1. Method A

2. Method B

(Method B)

**The Gambler's Fallacy (Kahneman & Tversky, 1972)**

A fair coin presents an equal probability of 50% for a Head or a Tail outcome.

Given that in three tosses of a fair coin, the outcome sequence was Tail, all three times (i.e., T-T-T), what would be the probability of each outcome in the next (fourth) toss:

Head ____ (50%)

Tail_____ (50%)

**Debt Account Aversion (Amar et al., 2010)**

Imagine that you have four different credit accounts with different balances, each of which has a different annual percentage rate (APR)

| Account | Debt balance | APR |
| --- | --- | --- |
| A | $2,830 | 2.5% |
| B | $3,476 | 2% |
| C | $5,080 | 3.5% |
| D | $7,200 | 3.25% |

Suppose that you have just received a $3,000 government stimulus rebate and that you have decided to use the entire rebate to pay off debt. How much would you allocate to each account?

Account A ________ (0%)

Account B ________ (0%)

Account C ________ (100%)

Account D ________ (0%)

(All money should be allocated to Account C – the account with the highest APR).

**The Hot Hand Effect (Gilovich, Tversky & Vallone, 1994)**

In tennis**,** an ace is a legal serve that is not touched by the receiver, which wins the point.

Player A is known for her excellent serves, and on average, she hits aces on 40% of her serves.

Imagine that in the game she is currently playing, Player A just hit 2 aces in a row. What is the probability that on her next serve, she will hit an ace?

Ace_____ (40%)

Not an Ace____ (60%)
